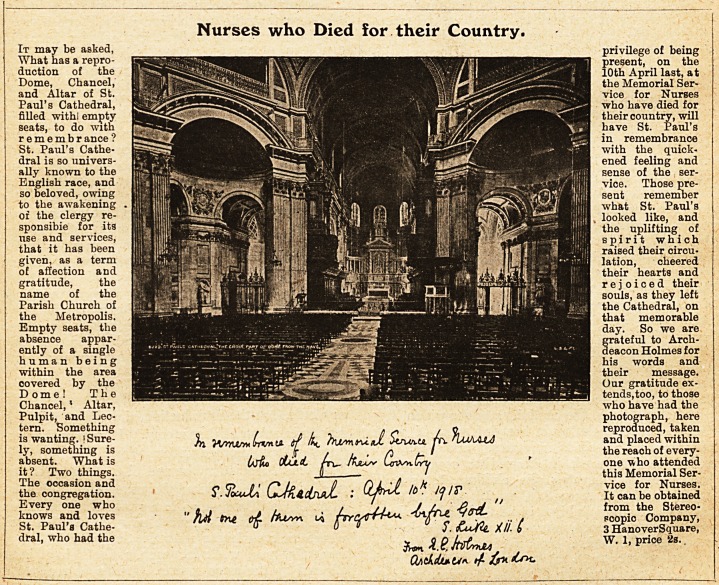# Round the Hospitals

**Published:** 1918-06-22

**Authors:** 


### ROUND THE HOSPITALS.

The Queen has graciously announced her willingness to accept as a silver wedding gift a "shower" of offerings for the wounded in the military and Red Cross hospitals. This and the gift of the City of London are to be the only exceptions to the rule made by herself and the King to decline all presents on the occasion of their silver wedding on July 6. Lady Lawley, G.B.E., St. James's Palace, S.W. 1., will receive and acknowledge all gifts, which should be marked "Silver Wedding Shower." They may be of any conceivable description, small or large, with the single proviso that they should be serviceable for the clothing, comfort, healing, or recreation of the men. Newfoundland has given a good lead to the Mother Country by already sending over 6,000 pairs of socks for the "Shower." Gifts from members of the nursing profession will, we surmise, prove particularly grateful to the Queen.

A good deal was said by Miss Haldane and others at the College of Nursing Conference on June 7 on the importance of receiving women of good education and broad views for State service. She believes that the College will do much to encourage such women to enter the nursing profession, by laying the foundation for shorter hours and better pay, but too little was said of the curriculum to be provided whereby university women might gain the inestimable advantage of hospital training without losing touch with larger interests. Dr. Hyslop Thomson, D.P.H., County Medical Officer of Health for Staffordshire, outlined some of the numerous forms of activity in which nurses must bear their part in the reconstructive period after the war, midwifery, school cases, tuberculosis, ophthalmia neonatorum, etc., all of which sometimes fell to the lot of one woman, and advocated post-graduate lectures to prepare nurses for such specialised departments. This expedient would not, in our judgment, fully meet the need.

"The Work and Necessary Qualifications of the School Nurse" were dealt with by Dr. C. J. Thomas, of the Department of Public Health, in a short paper full of illuminating facts. Both he and Dr. Jessie M. Campbell, who spoke on the "Tuberculosis Nurse," exposed the fact that qualifications needed by these special nurses called for important differentiation in their training. Dr. Campbell considers that were the hours on duty reduced to eight it would be possible for nurses to get part of their training outside the hospital, and so prepare for more extensive and special study in their third year. The most valuable and suggestive paper of the conference was contributed by Col. Thackray Parsons, R.A.M.C., on "The Nursing Profession in Relation to the proposed Ministry of Health." He outlined the changes likely to accrue to the nursing profession in the development of a State Nursing Service, and ended with an eloquent plea for the inclusion in one training-school of the entire training of the nurses. "The training-school," he said, "should be more than an institution for giving technical instruction; it should be a living body, of which the pupil feels herself to be a living part." "This sense of corporate life is absent in the case of divided training."

In the discussion which followed Col. Parsons' speech Miss Hamilton, matron of the Royal Hospital for Diseases of the Chest, Miss Hughes, Miss Gibson, and others took part. The centre of interest drifted away from the problem of preparing, within their own training-school, school tuberculosis and welfare nurses, health visitors, inspectors, and the like for the reconstructive work of the future, and settled on the more widely recognised problems connected with utilising special hospitals and the Smaller Poor-Law Unions for nurse-training. Miss Gibson's views, backed as they are by many decades of constructive work, were listened to with particular interest and respect. Miss Cox-Davies, in conclusion, paid a war tribute to the value of the Poor-Law nurses in the military hospitals.

The new departure at the Middlesex Hospital in inviting outside nurses to take a year's work in the cancer wing is likely to be equally serviceable to the training-school and to the nurses w[illegible] take the course. There is no doubt that the work of these wards demands trained nurses and is unsuited to probationers in their early stages. It is of a highly specialised nature. It develops the best qualities of a nurse as few other departments of nursing are able to do. But a certain amount of nursing experience is necessary before a probationer can benefit by it. It requires intelligent cooperation with surgeons who are carrying out the most delicate tests and experiments. Carried out as this experimental work has been at the Middlesex for the last generation, it affords an inspiring lesson in humanity. A current of high devotion to duty is set stirring in the wards where suffering is countered by science, and a fine spirit of gentleness and pity hovers over all—donors, surgeons, nurses—pledged to the deliverance of mankind from one of its worst plagues. To give a year to such work is to take part in a campaign wherein victory means the saving of more life than this war has ended. The opportunity now afforded to nurses whose training may have been of a limited character to share in it is bound to draw in just the class of women needed. The salary offered is from £40, with opportunities for self-improvement and a special certificate of competence in this branch of work at the conclusion of a year.

Miss Montgomery, lady superintendent of the Middlesex Hospital, is fully aware of the exacting demands made on the nurse's mentality by work in the cancer wing. Three hours off duty every day, half a day once a week, and a day once a month are liberal conditions of leave as the standard goes at present. But for this work, more than any other, the ideal is certainly one day in seven, and it is no secret that this is what the authorities aim at when the new scheme for the

**ROUND THE HOSPITALS—(*continued*).**

wing is in full working order. This reform demands additional nurses in the department, and could it be carried out there is no doubt that the strain on all the nurses would be appreciably lessened. It needs but that for nursing in the Wing to become a coveted service. The relief and added freshness which such a concession would impart to all engaged in this high national service would amply compensate for the extra cost, and the promise of sufficient extra nurses would, we believe, place it at once within reach.

At the annual meeting of the Royal British Nurses' Association last week Princess Christian bore welcome testimony that her niece, Princess Arthur of Connaught, was now engaged in nursing at St. Mary's Hospital, not only in the wards but also in the out-patient and casualty departments. Such practical services were frequently rendered in mediæval days by great ladies, though usually under cover of a religious order, and it is only a quasi-modern notion which shuts off a section of the community from the common labours and interests of their kind. None have done more than our own Royal Family to encourage a return to ancient simplicity in the service of humanity.

The opening of the Edith Cavell Nurses' Home at the London Hospital last week marks yet another advance in the standard of accommodation for nurses. The Nurses' Home at King's College Hospital, Denmark Hill, will still hold the palm, but visitors to the London will find, despite the site, that the architect and surveyor of the building have done admirably in their ability to use every inch of space for the nurses' convenience, and produce with all the wealth of practical detail a sense of harmony and proportion. Edith Cavell, shot by order of the German Governor of Belgium on October 12, 1915, was for five years a nurse at the London Hospital. This worthy memorial of her, crowned by the beautiful bust by Sir George Frampton which stands in the nurses' sitting-room, with stirring words by Mr. Asquith underneath, serves not only to keep her memory green in her own training-school, but acts as a reminder to all future generations of nurses of the heights to which their profession may lead them.

The Scottish Women's Hospitals is an organisation interesting to nurses for many reasons, and not least because it is one of the chosen objects for which the promoters of the Nation's Tribute to Nurses collected large sums of money. The story

### Nurses who Died for their Country.

It may be asked, What has a reproduction of the Dome, Chancel, and Altar of St. Paul's Cathedral, filled with empty seats, to do with remembrance? St. Paul's Cathedral is so universally known to the English race, and so beloved, owing to the awakening of the clergy responsibie for its use and services, that it has been given, as a term of affection and gratitude, the name of the Parish Church of the Metropolis. Empty seats, the absence apparently of a single human being within the area covered by the Dome! The Chancel, Altar, Pulpit, and Lectern. Something is wanting. Surely, something is absent. What is it? Two things. The occasion and the congregation. Every one who knows and loves St. Paul's Cathedral, who had the privilege of being present, on the 10th April last, at the Memorial Service for Nurses who have died for their country, will have St. Paul's in remembrance with the quickened feeling and sense of the service. Those present remember what St. Paul's looked like, and the uplifting of spirit which raised their circulation, cheered their hearts and rejoiced their souls, as they left the Cathedral, on that memorable day. So we are grateful to Archdeacon Holmes for his words and their message. Our gratitude extends, too, to those who have had the photograph, here reproduced, taken and placed within the reach of everyone who attended this Memorial Service for Nurses. It can be obtained from the Stereoscopic Company, 3 Hanover Square, W. 1, price 2s.

In remembrance of the Memorial Service for Nurses

Who died for their Country

S. Paul's Cathedral: April 10th 1918

"Not one of them is forgotten before God"

S. Luke XII. 6

From E. E. Holmes

Archdeacon of London

**ROUND THE HOSPITALS—(*continued*).**

of what Dr. Elsie Inglis accomplished for the Serbian Army with the units which she took over to that country is one of the most tragic yet glorious incidents of the war. Dr. Inglis took another unit to Russia and Roumania in the autumn of 1916, and, after undergoing great privations, brought it safely back to England a year later, but died in the act. Her skill and endurance, with her wonderful qualities as a leader and her tenderness towards the wounded, whom she set out to rescue from unspeakable sufferings and neglect, have set her very high among women. We learn with satisfaction that the London units of the Scottish Women's Hospitals have decided to commemorate this brave woman by endowing a Chair of Medicine at Belgrade when the University of the Serbian capital can be reconstituted after the war. The Lord Mayor is to preside at a meeting at the Mansion House to inaugurate the fund on July 5.

Letters which have appeared in our columns recently complaining of domestic difficulties through the absorption of so many workers in the various women's corps, make it evident that some new source of labour must be tapped for the supply of ward-maids and scrubbers, both in voluntary and Poor-Law hospitals. Would not the engagement of part-time workers, paid by the hour, meet the existing need? Lest this should be deemed a return to the deplorable "char" of the past, we would explain that a very different class of worker is aimed at and available. It is the younger women, soldiers' wives, women of good standing living in the neighbourhood of the institution, who could, we believe, be enlisted in the Hospital Service if care were taken to suit the conditions under which they live so as to make their hospital spell of work consistent with their home duties. Some could do early morning work if allowed to get home to breakfast and dispatch the children to school. Many could give from 8.30 to 12, or 1.30 to 5. Some who had no children could undertake parlour-work, serving meals, etc. Many are free in the evenings. There is a great waste of labour among women whose home duties do not nearly absorb all their time, and the Hospital Service, cleverly organised, many such women would reckon as an honour. Set before them in the right light it would be taken up distinctly as a patriotic duty. And no one is averse in these days from adding a few shillings to the weekly income. Such workers should be put at once into uniform, and graded according to length of service and efficiency.

A memorial tablet was unveiled on June 11 by the Bishop of Birmingham, in the chapel of the Queen's Hospital, Birmingham, to Miss Maude Amy Buckingham, formerly matron of the Queen's Hospital, and at the time of her death (Dec. 4, 1915) matron of the Hollymoor War Hospital. It is the gift of the sisters and nurses, past and present, trained at the Queen's, and represents in some small measure their affection and respect for their chief, whose influence in the training-school has left its mark for good on a generation of nurses. The service was taken by the chaplain of the institution, Captain the Rev. G. H. Moore, and the Bishop gave a beautiful address on the duties and privileges of women in war-time. An interesting feature of the occasion was the dedication for use in this chapel of three Bibles presented by Mr. Allen Edwards, one of the Bishop Hall Trustees.

The movement in favour of more adequate salaries for sisters and nurses is quietly gaining recruits. We learn that the authorities at the York County Hospital are raising probationers' salaries from £6, £12, £16, to £6, £18, £20. Sisters start at £40, with a war bonus of £5, and a rise to £50. Some idea of the necessary cost involved can be gained from the fact that the Royal Hampshire County Hospital, Winchester, has spent £550 on higher salaries during the past year, much of which has represented an improved scale of salary for the sisters, though a general increase in wages also has helped to swell the amount. Sisters and nurses, therefore, who may feel that this improvement only reflects the rise in prices, should remember that a higher salary has this advantage, as has been proved in the Colonies before now: that, though it is easier to spend because of the rise in the cost of living, it makes saving easier also.

**DO SO NOW**

After Monday, June 24th, you will not be able to get The Hospital unless you give your Newsagent a definite order.

**DO SO NOW**

We should be glad to know whether matrons find as a rule that the introduction of a slicing machine results in substantial economy. In one of the hospitals which afforded information to the British Hospitals Association the use of a slicing machine was said to have reduced the consumption of meat as much as fifteen stone in a week. This result is borne out by the experience of a military hospital where an even larger reduction, amounting to one meat diet per week for each patient, had been effected by the same means. If this be really the case, why is the use of this simple labour-saving apparatus not more general? We have too often seen meat hacked into the most unappetising fragments by the ordinary method of setting an inexpert person to manipulate a blunt knife, and we cannot conceive the cause of the prejudice in some quarters against mechanical aid.

We regret that, owing to an error in the official list supplied to us for our issue of June 15, page 230, Miss Worsley, Matron of the Children's Infirmary, Liverpool, was wrongly described as Matron of the Children's Infirmary, Edinburgh.

**For Viscount Knutsford's Reply to "Ierne's" Shylock's Article, see p. 253; Sir Arthur Stanley's Visit to Ireland, p. 254; Dr. Mercier and Asylum Workers, p. 249.**

## Figures and Tables

**Figure f1:**